# The First Highly Contiguous Genome Assembly for the Western Bluebird (*Sialia mexicana*)

**DOI:** 10.1093/gbe/evag219

**Published:** 2026-08-27

**Authors:** Carlos W Esperanza, Haley K Gee, Renée A Duckworth, Andrea D Schreier

**Affiliations:** Department of Animal Science, University of California Davis, Davis, CA, USA; Department of Ecology and Evolutionary Biology, University of Arizona, Tucson, AZ, USA; Department of Ecology and Evolutionary Biology, University of Arizona, Tucson, AZ, USA; Department of Animal Science, University of California Davis, Davis, CA, USA

**Keywords:** western bluebird, *Sialia mexicana*, reference genome, conservation genomics, population genomics

## Abstract

The western bluebird (*Sialia mexicana*) is a secondary cavity-nesting thrush that has experienced historical population declines, local extirpations, and more recent recoveries associated with nest box programs. Despite these regional successes, recent eBird estimates suggest continued range-wide declines and substantial geographic variation in population trajectories, making this species a useful system for future studies of demographic change, connectivity, and conservation genomics. However, genomic resources for western bluebirds remain limited, and no reference genome currently exists for any species in the genus *Sialia*. Here, we present the first high-quality de novo reference genome for *S. mexicana*. Using PacBio HiFi long-read sequencing from an adult female, we generated a highly contiguous, phased 1.3 Gb nuclear assembly with a contig N50 of 24.8 Mb and high BUSCO completeness of 98.3%. We annotated the nuclear genome using transcriptomic and protein evidence, identifying 16,656 protein-coding genes and 26,060 transcripts/protein isoforms. We also assembled a complete ∼16 kb mitochondrial genome from Illumina short-read data. This reference genome provides a foundational resource for future studies of population structure, genetic diversity, connectivity, demographic history, and adaptation in western bluebirds and related taxa.

Significance Western bluebirds (*Sialia mexicana*) have experienced historical declines, local extirpations, and human-mediated recolonization through nest box programs, yet the genomic consequences of these demographic changes remain largely unknown. This gap is important because genome-wide data are needed to evaluate population connectivity, genetic diversity, inbreeding, and adaptation across heterogeneous landscapes. Here, we present the first high-quality reference genome for *S. mexicana* and the first highly contiguous reference genome for the genus *Sialia*. This conspecific resource supports read mapping, variant discovery, gene annotation, comparative analyses, and future population genomic studies.

## Introduction

Extrinsic pressures that reduce population size, such as habitat loss, over-exploitation, increased competition from invasive species, and climate change, are major drivers of biodiversity loss worldwide (Bellard et al. 2022). Birds are particularly vulnerable to these pressures, with many species experiencing ongoing population declines globally and across North America (Rosenberg et al 2019; Lees et al. 2022). These declines provide an important context for investigating how the basic principles of population genetics shape the persistence of natural populations in changing environments. Demography and genetics, for example, interact at a fundamental level in determining extinction risk: small, isolated populations can experience inbreeding, potentially leading to reduced fitness, while genetic drift can randomly reduce the genetic variation that is needed for future adaptation (Lande 1988). Thus, characterizing patterns of genetic diversity and gene flow among populations undergoing decline and demographic change is necessary for predicting the future of vulnerable species. Reference genome assemblies provide a foundational map of the features and organization of an organism's genome, enabling genome-wide analyses of variation, population structure, and demographic history. Using a high-quality conspecific assembly can improve read mapping, variant discovery, and demographic inference by reducing biases that arise when genomic data are aligned to a more distantly related species (Bentley and Armstrong 2022).

**Fig. 1.**
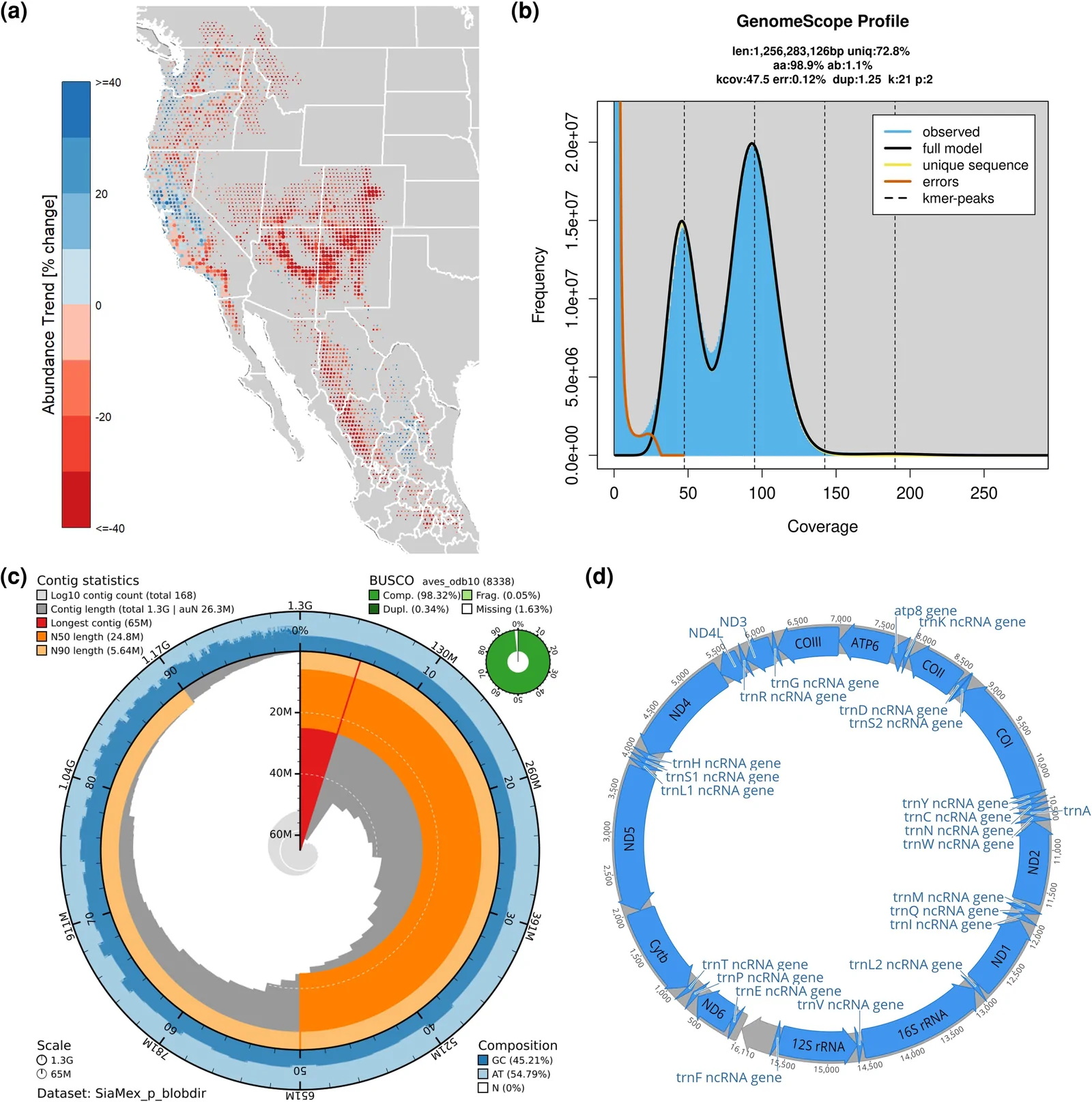
Contig-level genome assembly of *Sialia mexicana*. a) Estimated changes in western bluebird abundance from 2007 to 2022 across the species’ breeding range. Red areas indicate declining abundance, whereas blue areas indicate increasing abundance. Data from eBird Status and Trends, Cornell Lab of Ornithology (Fink et al. 2023). b) GenomeScope *k*-mer profile (*k* = 21) showing the estimated genome size, % heterozygosity, and % repetitive content. The histogram depicts a clear diploid *k*-mer profile with two distinct peaks representing heterozygous *k*-mers. c) Snail plot for haplotype 2 showing quality metrics (N50, N90, GC%) and BUSCO completeness. d) Complete mitochondrial assembly consisting of 16,110 bp, 13 protein-coding genes, 22 tRNAs, and 2 rRNAs.

The western bluebird (*Sialia mexicana*) provides an ideal system for studying the genomic consequences of demographic shifts and anthropogenic changes (Fig. S1). In western North America, the destruction of grasslands and logging of forests have been major factors contributing to the decline of terrestrial bird populations over the past century (DeSante and George 1994). Western bluebirds were once widely common in western North America but experienced substantial population declines, near extirpations, and elevational habitat shifts during the mid-20th century due to habitat fragmentation, interspecific competition, and pesticide exposure (Dawson and Bowles 1909; Bertrand and Scott 1979; Gilligan et al. 1994; Campbell et al. 1997; Duckworth and Badyaev 2007). Subsequent recolonization throughout parts of the species’ historical range was largely facilitated by nest box programs (Campbell et al. 1997; Purcell et al. 1997). However, data from eBird citizen science observations suggest that this species is still undergoing declines throughout most of its breeding range, with a median change in estimated abundance of 21.8% from 2007 to 2022 (Fig. 1a).

Despite the species’ history of decline, recolonization, and regionally variable demographic trends, genomic resources for this species remain limited. Previous genetic studies in *S. mexicana* have focused primarily on phylogenetic placement or behavioral traits, and no high-quality reference genome currently exists for any species in the genus *Sialia*. Generating a high-quality conspecific reference genome is therefore an essential first step for future population genomic analyses of genetic diversity, connectivity, hybridization dynamics, demographic history, and adaptation (Klicka et al. 2005; Charmantier et al. 2007; Ferree et al. 2008; Duckworth and Semenov 2017; Sauve et al. 2020; Moelling and Duckworth 2024; Veale et al. 2024). Here, we fill this resource gap by presenting the first high-quality de novo genome assembly for the western bluebird generated using PacBio HiFi long-read sequencing and annotated with protein and transcriptomic evidence. A complete mitochondrial genome was generated using whole genomes sequenced via Illumina short-read sequences. This genome provides a foundational resource for future studies of population structure, connectivity, demographic history, adaptation, and conservation genomics in this species and related taxa.

## Results and Discussion

### Nuclear Genome Assembly

PacBio HiFi sequencing generated 122.41 Gb of sequence data, corresponding to approximately 97.4× coverage based on the *k*-mer-estimated genome size (Fig. 1b; Table 1; Figs. S2 and S3). *K*-mer counting predicted a genome size of 1,256,283,126 bp, with 27.2% repetitive content and 1.1% heterozygosity (Fig. 1b). Assembly with Hifiasm produced two partially phased haplotype assemblies, arbitrarily labeled hap1 and hap2. After further post-assembly duplication purging, the final haplotype assemblies were 1.42 Gb and 1.30 Gb, respectively (Table 1; Table S1; Fig. S4). Hap2 was selected as the primary assembly because its total size was closer to the *k*-mer-estimated genome size, contained fewer contigs, and had a higher contig N50, higher *k*-mer QV completeness, and higher BUSCO completeness than hap1 (Table 1; Table S1; Fig. S5). The final primary assembly was 1,302,139,236 bp and consisted of 168 contigs, with a contig N50 of 24.78 Mb, a base-pair QV of 77.49, and 98.0% single copy BUSCOs using the aves_odb10 dataset (Table 1, Fig. 1c).

**Table 1:** Genome assembly and annotation metrics

HiFi read coverage	97.4×
Hifiasm assembly	Primary (hap2)
Assembly metrics	
Number of contigs	168
Contig N50 (bp)	24,780,003
Contig N90 (bp)	5,638,089
Contig NG50	26,963,476
Contig L50	17
Contig L90	66
auN	26,315,297.40
auNG	27,275,842.90
GC (%)	45.21
Largest contig	65,039,750
Size of final assembly	1,302,139,236
Base-pair QV	77.49
* k*-mer completeness (%)	85.84
BUSCO completeness (aves) *n* = 8338	C:98.3% [S:98.0%, D:0.3%], F:0.1%, M:1.6%
Annotation metrics	
Protein-coding genes	16,656
Transcripts	26,060
Predicted protein sequences	26,060
BUSCO completeness (aves) *n* = 8338	C:94.1% [S:93.5%, D:0.6%], F:0.5%, M:5.4%
CDS/exon features	326,452
Unique GO terms	9,512
Unique pathway annotations	15,240
Proteins with Swiss-Prot similarity	23,408/26,060 (89.8%)
Proteins with InterProScan matches	25,339/26,060 (97.2%)
Proteins with eggNOG annotations	24,075/26,060 (92.4%)

### Repetitive Content Annotation and Masking

Repeat annotation identified 368,840,933 bp of repetitive sequence in the primary assembly, representing 28.33% of the genome (Table S2). This estimate was broadly consistent with the *k*-mer-based repetitive content estimate of 27.2% (Fig. 1b). The largest fraction of annotated repeats consisted of unclassified elements, which accounted for 254,242,511 bp, or 19.52% of the assembly. Among classified repeats, retroelements represented 5.49% of the genome, including LTR elements (2.78%) and LINEs (2.71%), while SINEs were rare (0.002%) (Table S2). DNA transposons accounted for 3.31% of the assembly, with Tc1-IS630-Pogo elements representing the most abundant classified DNA transposon group. Small RNA-associated repeats made up only 0.10% of the genome. Compared with other published avian genomes, the western bluebird assembly falls among the more repeat-rich species included in our comparison and is similar in repetitive content to black redstart (*Phoenicurus ochruros*; 30.58%) and Bell's sparrow (*Artemisiospiza belli*; 31.2%) (Benham et al. 2024; Ghimire et al. 2025) (Fig. S6). In contrast, the closely related thrushes Bicknell's thrush (*Catharus bicknelli*) and gray-cheeked thrush (*C. minimus*) have substantially lower repetitive content despite belonging to a nearby passerine lineage (Termignoni-Garcia et al. 2022). This elevated repeat content is consistent with the relatively large genome size of western bluebird among the sampled avian genomes, supporting the broader observation that repeat content tends to increase with genome size in birds (Kapusta et al. 2017).

### Structural and Functional Gene Annotation

BRAKER3 predicted 16,656 protein-coding genes, representing 26,060 transcript/protein isoforms (Table 1). The final structural annotation contained 326,452 exon features, 326,452 CDS features, and 300,392 intron features. The protein and CDS FASTA files each contained 26,060 sequences. BUSCO analysis recovered 93.5% of aves_odb10 orthologues as single copy, with 0.6% duplicated, 0.5% fragmented, and 5.4% missing (Table 1).

Functional annotation using DIAMOND against Swiss-Prot assigned best-hit similarity annotations to 23,408 predicted protein isoforms (Table 1). InterProScan identified domain, family, or functional feature matches for 25,339 of 26,060 predicted proteins (97.2%), producing a broad set of InterPro, Gene Ontology, and pathway annotations (Table 1).

Orthology-based functional annotation with eggNOG-mapper assigned annotations to 24,075 predicted protein isoforms. These annotations included eggNOG orthologous groups, seed orthologs, COG functional categories, putative protein descriptions, preferred gene names, Gene Ontology terms, KEGG orthologs, KEGG pathway/module assignments, and Pfam annotations where available (Table S3).

### Mitochondrial Genome

The final draft *S. mexicana* mitochondrial genome assembly was 16,110 bp in length and included 13 protein-coding genes, 22 tRNA genes, and 2 ribosomal RNA genes, consistent with the typical structure of vertebrate mitogenomes (Fig. 1c).

## Materials and Methods

See Supplementary material for complete methods.

### Sample Collection, Library Prep, and Sequencing

A single adult female western bluebird was captured (USGS banding permit #23555, UC Davis IACUC protocol #24508) at the Putah Creek Nestbox Highway in California, United States, for PacBio HiFi genome sequencing. High molecular weight DNA extracted from blood was used for library preparation and long-read sequencing at the UC Davis DNA Technologies and Expression Analysis Core. Additional Illumina whole-genome resequencing data from 10 male western bluebirds collected between 2001 and 2014 from multiple western Montana populations (see Duckworth and Semenov 2017 and Duckworth et al. 2017 for detailed site descriptions) were used for mitochondrial genome assembly and validation analyses. For transcriptome-assisted genome annotation, 25 embryonic brain tissue samples were collected from western Montana nestbox populations during May–June 2024. RNA extracted from embryonic brain tissue was used for RNA sequencing and downstream evidence-guided gene annotation.

### Nuclear Genome Assembly and Assessment

*K*-mer frequency spectra were generated using meryl and visualized with GenomeScope2.0 to estimate genome characteristics prior to assembly (Ranallo-Benavidez et al. 2020). PacBio HiFi reads were assembled using Hifiasm v0.24.0 with built-in duplication purging (Cheng et al. 2021), producing a primary assembly and two phased haplotype assemblies. Remaining duplicated haplotigs were removed using purge_dups v1.2.5 (Guan et al. 2020). Assembly contamination was evaluated using Kraken2 and BLASTn (Camacho et al. 2009; Wood et al. 2019). Assembly statistics were calculated using QUAST (Gurevich et al. 2013), and genome completeness was assessed with BUSCO v5.7.1 against the aves_odb10 lineage dataset (Manni et al. 2021). Base-level accuracy and *k*-mer completeness were evaluated using Merqury (Rhie et al. 2021).

### Repeat Annotation and Gene Prediction

Repetitive elements were identified using a combined de novo and structure-based workflow with RepeatModeler v2.0.8 (Flynn et al. 2020) and EDTA (Ou et al. 2019). Repeat libraries generated by both pipelines were combined with RepBase sequences to construct a custom repeat library, and genome masking was performed using RepeatMasker v4.2.3 with the RMBlast search engine (Smit et al. 2013).

Protein-coding genes were predicted using BRAKER3 in ETP mode, integrating RNA-seq evidence, vertebrate protein homology evidence from OrthoDB v11, and ab initio gene prediction (Brůna et al. 2024; Gabriel et al. 2024). Predicted protein sequences were functionally annotated using complementary similarity-, domain-, and orthology-based approaches. DIAMOND v2.1.7 was used to search predicted proteins against Swiss-Prot (UniProt release 2024_07) with an *e*-value threshold of 1 × 10^−5^ (Buchfink et al. 2021). Conserved protein domains and associated functional annotations were identified using InterProScan v5.77-108.0 with InterPro accession lookup, Gene Ontology term assignment, and pathway annotation enabled (Jones et al. 2014; Blum et al. 2021). Orthology-based annotation was performed using eggNOG-mapper v2.1.13 in DIAMOND search mode against eggNOG v5.0.2 to assign orthologous groups, COG categories, Gene Ontology terms, and KEGG annotations (Cantalapiedra et al. 2021).

### Mitochondrial Genome Assembly and Annotation

Illumina resequencing reads were mapped to a reference avian mitochondrial genome (NCBI accession OR757050) using BWA v0.7.17 and SAMtools v1.19.2 to identify the individual with the highest mitochondrial coverage (Li and Durbin 2009; Danecek et al. 2021). The mitochondrial genome was assembled using GetOrganelle v1.7.7.1 (Jin et al. 2020) and annotated using MITOS2 through the Galaxy platform (Donath et al. 2019; The Galaxy Community 2024). Circular mitochondrial genome visualization was performed in Geneious Prime 2026.1.1.

## Supplementary Material

evag219_Supplementary_Data

## Data Availability

PacBio sequencing and data are available through NCBI BioProject PRJNA1471246, and RNA-seq data are available under PRJNA1479381. The mitochondrial genome is deposited under accession PZ467643. The final primary and alternate assemblies can be found on GenBank under accession numbers JCAOEU000000000 and JCAXWL000000000, respectively. Annotation files are available on Figshare at https://doi.org/10.6084/m9.figshare.32493435, and assembly code is available at https://github.com/CarlosEsperanza/WEBL_genome_assembly.git.
