# Supplementary material for: The First Highly Contiguous Genome Assembly for the Western Bluebird (Sialia mexicana)
Source: Genome Biol Evol. 2026 Aug 27;18(9):evag219. doi: 10.1093/gbe/evag219 (PMC13548056; doi:10.1093/gbe/evag219)
Supplement: Supplementary file 1 [file evag219_supplementary_data.docx]

The First Highly Contiguous Genome Assembly for the Western bluebird (*Sialia mexicana*)

**Supplementary Methods**

*Sample Collection and Tissue Processing*

For PacBio HiFi sequencing, a single adult female western bluebird was captured at the Putah Creek Nestbox Highway (Yolo County, CA) via mist-netting. Standard morphological measurements were recorded and approximately 50 μL of blood was collected from the brachial vein using a 26-gauge needle and stored in Queen’s Lysis buffer (Longmire et al., 1997). Samples were transported on ice and stored at −80°C until DNA extraction.

Embryonic brain tissue samples (n=25) used for RNA sequencing were collected from western Montana nestbox populations near Ovando, MT (see Duckworth et al., 2017 for site details) during May–June 2024. Eggs were maintained in a warmed transport container for up to two hours prior to laboratory processing. Embryos were dissected from eggs, decapitated, and whole brains were removed and flash frozen in isopentane supercooled with liquid nitrogen within 12 minutes of decapitation. Brain tissue was embedded in optimal cutting temperature compound (OCT), and right brain hemispheres were retained for RNA sequencing.

Brain tissues were homogenized by bead beating in QIAzol reagent (Qiagen) using 0.5 mm zirconium oxide beads supplemented with 1.0 mm beads to facilitate disruption of OCT-containing tissue. Homogenization was performed using a TissueLyser II (Qiagen) operated at 30 Hz under chilled conditions. RNA extraction followed the manufacturer’s recommended QIAzol protocol.

*HMW DNA Extraction, Library Prep, & Sequencing*

High-molecular-weight (HMW) genomic DNA (gDNA) was isolated from whole
blood preserved in Queen's lysis buffer (0.01 M Tris, 0.01 M NaCl,
0.01 M disodium-EDTA, 1.0% n-lauroylsarcosine, pH 8.0). A volume of
the lysate in Queen's buffer approximately equivalent to ~5 µL of the
original blood volume, was made up to 1 mL using Queen's buffer. This
mixture was treated with RNase A (20 µg/mL) at 37°C for 15 minutes,
followed by Proteinase K (100 µg/mL) digestion overnight (O/N) at room
temperature. The digested lysate was purified with an equal volume of
phenol/chloroform using Phase Lock Gel tubes. The DNA was precipitated
by adding a 0.4X volume of 5 M ammonium acetate and a 2X volume of
ice-cold ethanol. The resulting precipitate was washed with 70%
ethanol and resuspended in elution buffer (10 mM Tris, pH 8.0). DNA
purity was assessed by absorbance ratios (A60/A280 = 1.86 and
A260/A230 = 2.42) measured using a NanoDrop ND-1000 spectrophotometer
(Thermo Fisher Scientific, MA). The total DNA yield (55 µg) was
quantified using the QuantiFluor ONE dsDNA Dye assay (Promega, WI).
The size distribution of the HMW DNA was estimated using the Femto
Pulse system (Genomic DNA 165 kb kit, Agilent, CA), which revealed
that 95% of the total fragments were 150 kb or longer.

The HiFi SMRTbell library was constructed using the SMRTbell prep kit 3.0 (Pacific Biosciences, Menlo Park, CA; Cat. #102-182-700) according to the manufacturer's instructions with minor modifications. HMW gDNA was sheared to a target modal size of ~20 kb via automated repetitive pipetting using a CyBio FeliX liquid handling robot (Analytik Jena, Jena, Germany; cat. OL5015-24-100). The sheared DNA was concentrated using 1X SMRTbell cleanup beads, followed by repair and A-tailing (37°C for 60 minutes and at 65°C for 5 minutes), ligation of the SMRTbell adapters (20°C for 30 minutes), clean-up using 1X SMRTbell cleanup beads, nuclease treatment (37°C for 15 minutes), and final library clean-up using 1X SMRTbell cleanup beads. Size selection was performed on the SMRTbell library using the LightBench CF system from Yourgene Health for the removal of 12 kb and smaller fragments (Yourgene Health, Manchester, UK). The resulting ~22 kb average HiFi SMRTbell library was sequenced at the UC Davis DNA Technologies Core (Davis, CA) using one 25 M SMRT cell (Pacific Biosciences, Menlo Park, CA; Cat #102-202-200) on the Revio system with SPRQ sequencing chemistry and a 30-hour movie time.

*Genome Assembly and Quality Assessment*

K-mer frequency spectra (k = 21) were generated using meryl and visualized with GenomeScope2.0 (Ranallo-Benavidez et al., 2020) to estimate genome size, heterozygosity, and repetitive content. Hifiasm v0.24.0 was run with default parameters and built-in duplication purging to generate a primary pseudo-haploid assembly and two phased haplotype assemblies (Cheng et al., 2022). Downstream analyses focused on the haplotype-resolved assemblies to reduce redundancy associated with collapsed haplotypes.

Remaining duplicated haplotigs were removed using purge_dups v1.2.5 with empirically determined cutoff values (Guan et al., 2020). Contamination screening was performed using Kraken2 and BLASTn searches (Camacho et al., 2009; Wood et al., 2019), and non-avian contigs were removed from the assemblies. Raw HiFi reads were mapped back to each assembly using minimap2 v2.2.1 (Li et al., 2018) to assess read support and assembly consistency.

Assembly contiguity statistics including N50, NG50, auN, and auNG were calculated using QUAST (Gurevich et al., 2013). Genome completeness was assessed using BUSCO v5.7.1 against the aves_odb10 ortholog database containing 8,330 genes (Manni et al., 2021). Base-level accuracy and k-mer completeness were estimated using Merqury (Rhie et al., 2020).

*Repeat Annotation and Masking*

RepeatModeler v2.0.8 was used for de novo repeat family discovery using the RMBlast search engine (Flynn et al., 2020). EDTA was additionally used to identify structurally recognizable transposable elements, including LTR retrotransposons, terminal inverted repeat elements, MITEs, and Helitrons (Ou et al., 2019). Repeat libraries generated by RepeatModeler2 and EDTA were combined with RepBase repeat sequences to create a comprehensive custom repeat library. Repeat masking and annotation were performed using RepeatMasker v4.2.3 with RMBlast (Smit et al., 2013).

*Gene Annotation and Functional Annotation*

Gene prediction and annotation were performed using BRAKER3 in ETP mode, integrating RNA-seq data, protein homology evidence, and ab initio prediction (Brůna et al., 2024; Gabriel et al., 2024). Embryonic brain RNA-seq reads were aligned to the repeat-masked genome assembly and used alongside the OrthoDB v11 vertebrate protein database to guide gene prediction through the GeneMark-ETP and AUGUSTUS pipelines. Final evidence-supported gene models were selected and integrated using TSEBRA.

Functional annotation of predicted proteins was performed using complementary similarity-based, domain-based, and orthology-based approaches. Predicted protein sequences were searched against the Swiss-Prot database (UniProt release 2024_07) using DIAMOND v2.1.7 BLASTp with an e-value threshold of 1 × 10⁻⁵ to identify homologous protein sequences and assign putative gene functions (Buchfink et al., 2021). Conserved protein domains, families, and functional signatures were identified using InterProScan v5.77-108.0 with InterPro accession lookup, Gene Ontology term assignment, and pathway annotation enabled (Jones et al., 2014; Blum et al., 2021).

Predicted protein sequences were additionally annotated using eggNOG-mapper v2.1.13 in DIAMOND search mode against the eggNOG v5.0.2 database. eggNOG-mapper was used to assign orthology-based functional annotations, including seed orthologs, eggNOG orthologous groups, taxonomic annotation levels, COG functional categories, predicted protein descriptions, preferred gene names, Gene Ontology terms, enzyme commission numbers, KEGG orthologs, KEGG pathways/modules, and Pfam annotations where available.

Functional annotations from Swiss-Prot, InterProScan, and eggNOG-mapper were retained as transcript-level annotations linked to BRAKER3 transcript identifiers. BRAKER3 gene and transcript IDs were preserved as stable annotation identifiers, while putative gene names, product descriptions, domain annotations, Gene Ontology terms, pathway annotations, KEGG annotations, eggNOG orthologous groups, and COG categories were compiled into integrated transcript-level annotation tables. Gene model identifiers from BRAKER3 were retained as stable annotation IDs, while putative gene names, product descriptions, InterPro accessions, Gene Ontology terms, pathway annotations, KEGG annotations, eggNOG orthologous groups, and COG categories were integrated into transcript-level annotation tables and annotated GFF3, protein FASTA, and CDS FASTA files.

*Mitochondrial Genome Assembly and Annotation*

To identify the optimal sample for mitochondrial genome assembly, Illumina resequencing reads from 10 western bluebird individuals were mapped against the avian mitochondrial reference genome (accession number OR757050) using BWA v0.7.17 (Li & Durbin, 2009). Alignment processing and coverage statistics were generated using SAMtools v1.19.2 (Danecek et al., 2021), including mean coverage depth, mapped read counts, and breadth of mitochondrial genome coverage.

The individual with the highest mitochondrial coverage was selected for de novo mitochondrial genome assembly using GetOrganelle v1.7.7.1 in animal mitochondrial mode (-F animal_mt) with multiple k-mer sizes (21, 45, 65, 85, 105, and 127) (Jin et al., 2020). Read reduction limits were disabled to maximize mitochondrial read retention during assembly.

The resulting mitochondrial assembly was annotated using MITOS2 through the Galaxy platform (Donath et al., 2019; The Galaxy Community, 2024). Annotation files were converted from GFF to GenBank format for downstream visualization and inspection. Circular mitochondrial genome maps were generated using Geneious Prime 2026.1.1.

**Supplementary Figures & Tables**

Supplemental Figure 1: Breeding male (right) and female (left) Western bluebirds. Western bluebirds are sexually dimorphic, with males showing a more vibrant blue color on their head, wings, and back, and a darker rust color on their breasts. Photo taken by Peimeng Xu in Oceanside, CA.

Supplemental Figure 2: Read length distribution of Raw Pac Bio Hifi reads.

Supplementary Figure 3: Quality score distribution of raw HiFi reads.

Supplementary Figure 4: K-mer copy number spectrum for the *Sialia mexicana* haplotype assembly. K-mer multiplicity spectra were generated to evaluate assembly completeness and redundancy. The x-axis shows k-mer multiplicity, and the y-axis shows k-mer counts. Colors indicate the number of times each k-mer from the assembly is represented, with gray representing read-only k-mers not present in the assembly and red representing single-copy assembly k-mers. The major assembly-supported peaks correspond to heterozygous and homozygous sequence content, while the low proportion of higher-copy k-mers suggests limited residual duplication in the purged haplotype assembly.

Supplementary Figure 5: Snail plot depicting the overall assembly statistics, bp composition, and BUSCO score for the alternate (hap1) assembly.

Figure S6. Genome size and repetitive content across selected avian genomes. Genome assembly size (x-axis) is plotted against the percentage of each assembly annotated as repetitive sequence. The western bluebird assembly contains 28.33% repetitive sequence and falls among the larger, more repeat-rich genomes included in this comparison, similar to Black redstart (*Phoenicurus ochruros*) and Bell’s sparrow (*Artemisiospiza belli*). In contrast, the closely related thrushes Bicknell’s thrush (*Catharus bicknelli*) and gray-cheeked thrush (*Catharus minimus*) have substantially lower repetitive content despite belonging to a nearby passerine lineage. The fitted line illustrates the general positive association between genome size and repeat content across the sampled species. This comparison is intended as descriptive context rather than a phylogenetically controlled analysis. Genome size and repetitive data from Termigoni-Garcia et al., 2022, and Ghimire et al., 2025.

| **Supplementary Table 1:**  **Alternate genome assembly metrics** | |
| --- | --- |
| Hifiasm assembly | **Alternate (hap1)** |
| **Assembly metrics** | − |
| Number of contigs | 202 |
| Contig N50 (bp) | 19977370 |
| Contig N90 (bp) | 5451966 |
| Contig NG50 | 22516635 |
| Contig L50 | 20 |
| Contig L90 | 72 |
| auN | 29200468.2 |
| auNG | 32896025.6 |
| GC (%) | 47.04 |
| Largest contig | 90832550 |
| Size of final assembly | 1415276002 |
| Base pair QV | 74.29 |
| *k*-mer completeness (%) | 82.51 |
| BUSCO completeness (aves) n=8338 | C:95.6% [S:95.2%, D:0.4%], F:0.1%, M:4.4% |

| **Supplementary Table 2**  Summary of repetitive element composition in the *Sialia mexicana* genome assembly identified using a combined *de novo* and structure-based repeat annotation workflow integrating RepeatModeler, EDTA, RepBase, and RepeatMasker. | | | |
| --- | --- | --- | --- |
|  | **Number of elements** | **Length (bp)** | **Percentage** |
| **Total interspersed repeats** |  | 368840933 | 28.33 |
| **Retroelements** | 193274 | 71489473 | 5.49 |
| SINEs | 374 | 30692 | 0.002 |
| LINES | 75128 | 35252496 | 2.71 |
| L2/CR1/Rex | 74968 | 35202532 | 2.7 |
| R2/R4/NeSL | 160 | 49964 | 0.004 |
| LTR elements | 117772 | 36206285 | 2.78 |
| **Ty1/Copia** | 24 | 34360 | 0.003 |
| Gypsy/DIRS1 | 5869 | 3341637 | 0.26 |
| Retroviral | 84604 | 28586277 | 2.2 |
| **DNA transposons** | 154882 | 43108949 | 3.31 |
| Hobo-activator | 59 | 16824 | 0.001 |
| Tc1-IS630-Pogo | 3020 | 2605100 | 0.2 |
| Small RNA | 1054 | 1280574 | 0.1 |
| **Unclassified** | 401086 | 254242511 | 19.52 |
|  |  |  |  |

| **Supplementary Table 3**  **Distribution of InterProScan match rows by analysis source.** Counts represent the number of InterProScan match rows assigned to predicted *Sialia mexicana* protein sequences by each InterProScan component database or analysis method, including MobiDBLite, Pfam, SMART, Gene3D, ProSiteProfiles, FunFam, SUPERFAMILY, PRINTS, CDD, PANTHER, Coils, ProSitePatterns, NCBIfam, PIRSF, Hamap, SFLD, and AntiFam. | |
| --- | --- |
| **InterProScan analysis** | **Match rows** |
| MobiDBLite | 120,762 |
| Pfam | 64,538 |
| SMART | 56,310 |
| Gene3D | 54,299 |
| ProSiteProfiles | 46,458 |
| FunFam | 42,912 |
| SUPERFAMILY | 41,829 |
| PRINTS | 39,830 |
| CDD | 26,665 |
| PANTHER | 23,577 |
| Coils | 20,908 |
| ProSitePatterns | 16,455 |
| NCBIfam | 2,255 |
| PIRSF | 1,974 |
| Hamap | 512 |
| SFLD | 439 |
| AntiFam | 1 |

**Work Cited**

Benham, P. M. *et al*. Remarkably High Repeat Content in the Genomes of Sparrows: The Importance of Genome Assembly Completeness for Transposable Element Discovery. *Genome Biol. Evol.* **16**, evae067 (2024).

Blum M, Chang HY, Chuguransky S, Grego T, Kandasaamy S, Mitchell A, Nuka G, Paysan-Lafosse T, Qureshi M, Raj S, Richardson L, Salazar GA, Williams L, Bork P, Bridge A, Gough J, Haft DH, Letunic I, Marchler-Bauer A, Mi H, Natale DA, Necci M, Orengo CA, Pandurangan AP, Rivoire C, Sigrist CJA, Sillitoe I, Thanki N, Thomas PD, Tosatto SCE, Wu CH, Bateman A, Finn RD. The InterPro protein families and domains database: 20 years on. Nucleic Acids Res. 2021 Jan 8;49(D1):D344-D354. doi: 10.1093/nar/gkaa977. PMID: 33156333; PMCID: PMC7778928.

Brůna T, Lomsadze A, Borodovsky M. GeneMark-ETP significantly improves the accuracy of automatic annotation of large eukaryotic genomes. Genome Res. 2024 Jun 25;34(5):757-768. doi: 10.1101/gr.278373.123. PMID: 38866548; PMCID: PMC11216313.

Buchfink BJ, Reuter K, Drost HG, "Sensitive protein alignments at tree-of-life scale using DIAMOND", *Nature Methods* **18**, 366–368 (2021). [doi:10.1038/s41592-021-01101-x](https://doi.org/10.1038/s41592-021-01101-x).

Camacho, C., Coulouris, G., Avagyan, V., Ma, N., Papadopoulos, J., Bealer, K., & Madden, T. L. (2009). BLAST+: architecture and applications. *BMC bioinformatics*, *10*(1), 421.

Cheng, H., Jarvis, E. D., Fedrigo, O., Koepfli, K. P., Urban, L., Gemmell, N. J., & Li, H. (2022). Haplotype-resolved assembly of diploid genomes without parental data. Nature Biotechnology, 40(9), 1332-1335.

Danecek P, Bonfield JK, Liddle J, Marshall J, Ohan V, Pollard MO, Whitwham A, Keane T, McCarthy SA, Davies RM, Li H. Twelve years of SAMtools and BCFtools. Gigascience. 2021 Feb 16;10(2):giab008. doi: 10.1093/gigascience/giab008. PMID: 33590861; PMCID: PMC7931819.

Duckworth, R.A., Hallinger, K.K., Hall, N., Potticary, A.L., 2017. Switch to a novel breeding resource influences coexistence of two passerine birds. Frontiers in Ecology and Evolution 5, 72.

Donath, A., Jühling, F., Al-Arab, M., Bernhart, S. H., Reinhardt, F., Stadler, P. F., Middendorf, M., & Bernt, M. (2019). Improved annotation of protein-coding genes boundaries in metazoan mitochondrial genomes. *Nucleic Acids Research*, *47*(20), 10543–10552. <https://doi.org/10.1093/nar/gkz833>

Flynn JM, Hubley R, Goubert C, Rosen J, Clark AG, Feschotte C, Smit AF. RepeatModeler2 for automated genomic discovery of transposable element families. Proc Natl Acad Sci U S A. 2020 Apr 28;117(17):9451-9457. doi: 10.1073/pnas.1921046117. Epub 2020 Apr 16. PMID: 32300014; PMCID: PMC7196820.

Gabriel L, Brůna T, Hoff KJ, Ebel M, Lomsadze A, Borodovsky M, Stanke M. BRAKER3: Fully automated genome annotation using RNA-seq and protein evidence with GeneMark-ETP, AUGUSTUS and TSEBRA. bioRxiv [Preprint]. 2024 Feb 29:2023.06.10.544449.

Ghimire, P., Wang, N., Hu, Y. *et al.* A complete reference genome assembly and annotation of the Black Redstart (***Phoenicurus ochruros***). *Sci Data* **12**, 1885 (2025). https://doi.org/10.1038/s41597-025-06156-5

Guan, D., McCarthy, S. A., Wood, J., Howe, K., Wang, Y., & Durbin, R. (2020). Identifying and removing haplotypic duplication in primary genome assemblies. *Bioinformatics*, *36*(9), 2896-2898.

Gurevich A, Saveliev V, Vyahhi N, Tesler G. QUAST: quality assessment tool for genome assemblies. Bioinformatics. 2013 Apr 15;29(8):1072-5. doi: 10.1093/bioinformatics/btt086. Epub 2013 Feb 19. PMID: 23422339; PMCID: PMC3624806.

Jin, J.J., Wen-Bin Yu*, Jun-Bo Yang, Yu Song, Claude W. dePamphilis, Ting-Shuang Yi, De-Zhu Li. GetOrganelle: a fast and versatile toolkit for accurate de novo assembly of organelle genomes. Genome Biology 21, 241 (2020). <https://doi.org/10.1186/s13059-020-02154-5>

Jones P, Binns D, Chang HY, Fraser M, Li W, McAnulla C, McWilliam H, Maslen J, Mitchell A, Nuka G, Pesseat S, Quinn AF, Sangrador-Vegas A, Scheremetjew M, Yong SY, Lopez R, Hunter S. InterProScan 5: genome-scale protein function classification. Bioinformatics. 2014 May 1;30(9):1236-40. doi: 10.1093/bioinformatics/btu031. Epub 2014 Jan 21. PMID: 24451626; PMCID: PMC3998142.

Li, H. Minimap2: pairwise alignment for nucleotide sequences, *Bioinformatics*, Volume 34, Issue 18, September 2018, Pages 3094-3100, <https://doi.org/10.1093/bioinformatics/bty191>

Li H. and Durbin R. (2009) Fast and accurate short read alignment with Burrows-Wheeler transform. Bioinformatics, **25**, 1754-1760. [PMID: [19451168](http://www.ncbi.nlm.nih.gov/pubmed/19451168)]. (if you use the BWA-backtrack algorithm).

Longmire, J., M. Maltbie, and R. J. Baker. 1997. Use of ‘Lysis Buffer’ in DNA Isolation and its Implication for Museum Collections. Museum of Texas Tech University.

Manni M, Berkeley MR, Seppey M, Simão FA, Zdobnov EM. BUSCO Update: Novel and Streamlined Workflows along with Broader and Deeper Phylogenetic Coverage for Scoring of Eukaryotic, Prokaryotic, and Viral Genomes. Mol Biol Evol. 2021 Sep 27;38(10):4647-4654. doi: 10.1093/molbev/msab199. PMID: 34320186; PMCID: PMC8476166.

Ou S, Su W, Liao Y, Chougule K, Agda JRA, Hellinga AJ, Lugo CSB, Elliott TA, Ware D, Peterson T, Jiang N, Hirsch CN, Hufford MB. Benchmarking transposable element annotation methods for creation of a streamlined, comprehensive pipeline. Genome Biol. 2019 Dec 16;20(1):275. doi: 10.1186/s13059-019-1905-y. PMID: 31843001; PMCID: PMC6913007.

Ranallo-Benavidez TR, Jaron KS, Schatz MC. GenomeScope 2.0 and Smudgeplot for reference-free profiling of polyploid genomes. Nat Commun. 2020:11:1. doi:10.1038/s41467-020-14998-3

Rhie, A., McCarthy, S.A., Fedrigo, O. et al. Towards complete and error-free genome assemblies of all vertebrate species. Nature 592, 737–746 (2021). <https://doi.org/10.1038/s41586-021-03451-0>

Smit Arian F. A., Hubley Robert, and Green Philip. RepeatMasker Home Page, 2013. [http://www.repeatmasker.org/’](http://www.repeatmasker.org/)

Termignoni-Garcia, F., Kirchman, J. J., Clark, J., & Edwards, S. V. (2022). Comparative Population Genomics of Cryptic Speciation and Adaptive Divergence in Bicknell's and Gray-Cheeked Thrushes (Aves: *Catharus bicknelli* and *Catharus minimus*). *Genome biology and evolution*, *14*(1), evab255. https://doi.org/10.1093/gbe/evab255

Wood, D. E., Lu, J., & Langmead, B. (2019). Improved metagenomic analysis with Kraken 2. *Genome biology*, *20*(1), 257.
